# Increasing the risk of spontaneous abortion and major malformations in newborns following use of serotonin reuptake inhibitors during pregnancy: A systematic review and updated meta-analysis

**DOI:** 10.1186/2008-2231-20-75

**Published:** 2012-11-01

**Authors:** Shekoufeh Nikfar, Roja Rahimi, Narjes Hendoiee, Mohammad Abdollahi

**Affiliations:** 1 Food and Drug Laboratory Research Center, Food and Drug Organisation, Tehran, Iran; 2 Department of Pharmacoeconomics and Pharmaceutical Administration, Faculty of Pharmacy, Tehran University of Medical Sciences, Tehran, Iran; 3 Department of Toxicology and Pharmacology, Faculty of Pharmacy, and Pharmaceutical Sciences Research Center, Tehran University of Medical Sciences, Tehran, Iran; 4 Department of Traditional Pharmacy, Faculty of Traditional Medicine, Tehran University of Medical Sciences, Tehran, Iran; 5 Department of Clinical Pharmacy, Faculty of Pharmacy, Mazandaran University of Medical Sciences, Sari, Iran

**Keywords:** Selective serotonin reuptake inhibitors (SSRIs), Pregnancy outcome, Meta-analysis, Evidence-based medicine, Malformation, Systematic review

## Abstract

Selective serotonin reuptake inhibitors (SSRIs) are the most frequently used antidepressants during pregnancy. There are conflicting results about their influence on pregnancy outcomes. The goal of this study was to update our previous meta-analysis about pregnancy outcomes following exposure to SSRIs. For this purpose, all relevant databases were searched from 1990 to March 2012 for studies investigating the pregnancy outcomes following exposure to any therapeutic dosage of any SSRI (fluoxetine, paroxetine, citalopram, escitalopram, sertraline, fluvoxamine) during pregnancy. Types of outcome investigated were spontaneous abortion, major malformations, cardiovascular malformations, and minor malformations. A total of 25 studies met our criteria and were included in the meta-analysis. The odds ratio (OD) values are 1.87 (95% CI: 1.5 to 2.33, P< 0.0001) for spontaneous abortion, 1.272 (95% CI: 1.098 to 1.474, P = 0.0014) for major malformations, 1.192 (95% CI: 0.39 to 3.644, P= 0.7578) for cardiovascular malformations, and 1.36 (95% CI: 0.61 to 3.04, P= 0.4498) for minor malformations. The results demonstrated that SSRIs increase the risk of spontaneous abortion and major malformations during pregnancy while they don’t increase the risk of cardiovascular malformations and minor malformations. Our previous meta-analysis only showed an increase in the risk of spontaneous abortion following the use of SSRIs during pregnancy. This might be due to increase in the number of studies included or addition of two new SSRIs (citalopram and escitalopram). The message to researchers is to try considering SSRIs individually during pregnancy to reduce heterogeneity, although all are aware of inevitable limitations to study on pregnant mothers.

## Introduction

Evidences show that there is an increase in psychiatric disorders particularly depression and anxiety during pregnancy [1,2]. Although the pathogenesis is unknown, hormonal dysregulation, abnormalities in hypothalamic-pituitary-adrenal axis activity, and the contributions of genetics and epigenetics seems playing key roles in the development of perinatal mood disorders [3]. Women with depression during pregnancy are at increased risk for premature delivery, low birth weight, and postpartum depression [4,5].

The estimate is that up to 13% of all pregnant women use an at least one antidepressant during pregnancy. Selective serotonin reuptake inhibitors (SSRIs) are the first-line, most frequently used antidepressants among pregnant women [3]. However, some studies demonstrated adverse pregnancy outcome following exposure to SSRIs. Results from our recent meta-analysis published in 2006 revealed that SSRIs do not increase the risk of major cardiovascular and minor malformations but do increase the risk of spontaneous abortion [6]. In the present study we have updated our previous meta-analysis by including more studies published in the recent 6 years about the effects of SSRIs on pregnancy outcomes.

## Methods

### Data sources

Scopus, PubMed, Web of Science, and Cochrane Central Register of Controlled Trials (CCRCT) were searched for studies that investigated the effect of SSRIs on pregnancy outcomes of depressive women. Data were collected for the years 1990 to 2012 (up to March). The search terms were “serotonin reuptake inhibitors” and “pregnancy”, “birth outcome”, or “obstetrical outcome”. For PubMed, all relevant MeSH terms were used. For Web of Science and CCRCT, the same entry terms including their abbreviations were applied. The final queries were validated by manual review and matching results. The reference lists from retrieved articles were manually reviewed for finding additional applicable studies.

### Study selection

All controlled studies that investigated the effect of SSRIs on pregnancy outcomes were considered. Spontaneous abortion, major malformations, minor malformations, and cardiovascular malformations were the key outcomes of interest. Major malformations are all singular and combined structural defects, syndromes, sequences, and associations. Minor malformations include small structural developmental disturbances that do not impair viability and do not need to be treated.

Studies and abstracts that were presented at meetings were also considered. Two reviewers independently examined the title and abstract of each article to eliminate duplicates, reviews, case studies, uncontrolled trials, trials did not have desired outcomes, and trials published in languages other than English. Reviewers independently extracted data on type of study, therapeutic regimens, time of exposure, and outcome measures. Disagreements were resolved by consensus.

### Statistical analysis

Data from selected studies were extracted in the form of 2×2 Tables. Included studies were weighted and pooled. The data were analyzed using StatsDirect software version 2.7.8. Odds ratio (OR) and 95% confidence intervals (95% CI) were calculated using the Mantel-Haenszel, Robins-Breslow-Greenland and Der Simonian-Laird methods. The Cochran Q test was used to test heterogeneity. In case of heterogeneity or probability of few included studies in meta-analysis, the random effects for individual and summary of effect size for weighted mean difference was applied. Funnel plot analysis was used as publication bias indicator.

## Results

**Figure 1:**
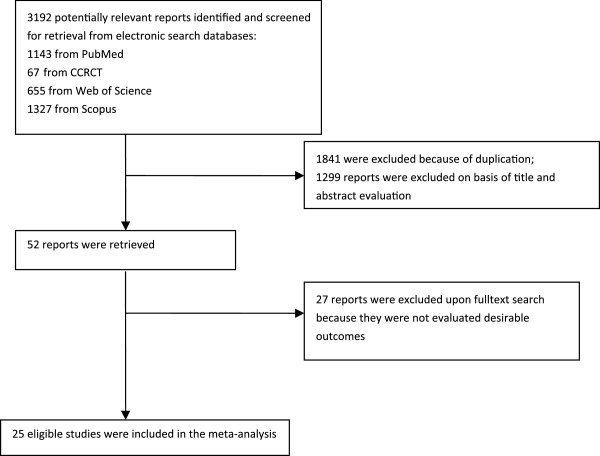
Flow diagram of the study selection process.

The electronic searches yielded 3192 items; 1143 from PubMed, 67 from CCRCT, 655 from Web of Science, and 1327 from Scopus. Of these, 52 trials were scrutinized in full text and 25trials [7-31] were included in the analysis (Figure 1). Type of study, SSRI subclass, and time of exposure for each study are presented in Table 1.

**Table 1:** **Characteristics and results of studies included in meta**-**analysis**

**Study**	**Drug**	**Type**	**Mean maternal age**	**Time of Ex**	**Spontaneous abortion**		**Major Mal**		**Cardiovascular Mal**		**Minor Mal**	
					**Ex**	**Non**-**Ex**	**Ex**	**Non**-**Ex**	**Ex**	**Non**-**Ex**	**Ex**	**Non**-**Ex**
Nordeng et al. 2012 [7]	Citalopram Escitalopram Paroxetine Fluoxetine Sertraline Fluvoxamine	Prospective cohort	ND	First trimester	-	-	12/462	1550/61648	6/462	541/61648	13/462	1279/61648
Malm et al. 2011 [8]	Citalopram Escitalopram Paroxetine Fluoxetine Sertraline Fluvoxamine	Retrospective cohort	29.6	First trimester	-	-	303/6976	22305/623402	116/6976	8137/623402	-	-
Colvin et al. 2011 [9]	Citalopram Paroxetine Fluoxetine Sertraline		30.2	First trimester	-	-	115/2701	3834/94561	30/2701	661/94561	30/6976	737/623402
Kornum et al. 2010 [10]	paroxetine fluoxetine citalopram escitalopram sertraline	Prospective	30.15	First trimester	-	-	-	-	26/2062	1403/213712	-	-
Merlob et al. 2009 [11]	Paroxetine Fluoxetine Citalopram Escitalopram Sertraline Fluvoxamine Venlafaxine	Prospective	ND	First trimester	-	-	8/235	1083/67636	8/235	1083/67636	-	-
Wichman et al. 2009 [12]	Paroxetine Fluoxetine Citalopram Escitalopram Sertraline Venlafaxine	Retrospective cohort	ND	Throughout	-	-	-	-	3/808	205/24406	-	-
Einarson et al. 2009 [13]	Paroxetine Fluoxetine Citalopram Sertraline Fluvoxamine	Prospective cohort	ND	First trimester	-	-	19/506	25/928	-	-	-	-
Diav-Citrin et al. 2008 [14]	Paroxetine Fluoxetine	Prospective Cohort	31.5	First trimester	-	-	30/601	34/1359	14/601	8/1359	-	-
Oberlander et al. 2008 [15]	Paroxetine Fluoxetine Citalopram Sertraline Fluvoxamine	Retrospective cohort	29.6	First trimester	-	-	75/2459	3369/107320	18/2459	512/107320	-	-
Einarson et al. 2008 [16]	Paroxetine	Retrospective cohort	ND	Throughout	-	-	-	-	9/1174	9/1174	-	-
Källén et al. 2007 [17]	Paroxetine Fluoxetine Citalopram Escitalopram Sertraline Fluvoxamine	Retrospective cohort	ND	First trimester	-	-	-	-	78/6555	11367/873876	-	-
Wen et al. 2006 [18]	Paroxetine Fluoxetine Citalopram Sertraline Fluvoxamine	Retrospective	ND	Throughout	-	-	20/961	76/3861	-	-	35/961	133/3861
Wogelius et al. 2006 [19]	ND, any SSRI	Retrospective cohort	ND	First trimester	-	-	51/1051	5112/150780	-	-	-	-
Vial et al. 2006 [20]	Paroxetine	Prospective cohort	31	First trimester	80/683	31/683	12/535	10/631	3/535	3/631	1/535	1/631
Sivojelezova et al. 2005 [21]	Paroxetine Fluoxetine Citalopram Sertraline	Prospective cohort	31.9	First trimester	27/264	13/132	4/223	1/118	2/223	-	-	-
Malm et al. 2005 [22]	Paroxetine Fluoxetine Citalopram Sertraline Fluvoxamine	Retrospective cohort	30	Throughout	-	-	75/1767	62/1779	-	-	-	-
Casper et al. 2003 [23]	Paroxetine Fluoxetine Sertraline Fluvoxamine	Prospective cohort	35.7	Throughout	-	-	1/31	1/13	1/31	0/13	24/31	7/13
Costei et al. 2002 [24]	Paroxetine	Prospective, controlled cohort	32.9	First trimester	-	-	0/27	0/27	0/27	0/27	-	-
Diav-Citrin et al. 2002 [25]	Paroxetine	Prospective cohort	31	Throughout	29/236	44/629	7/196	12/580	-	-	-	-
Simon et al. 2002 [26]	Paroxetine Fluoxetine Sertraline Fluvoxamine		ND	Throughout	-	-	12/185	9/185	1/185	2/185	-	-
Einarson et al. 2001 [27]	Paroxetine Fluoxetine Sertraline Fluvoxamine	Prospective controlled cohort	ND	First trimester	16/150	11/150	3/124	1/137	1/124	1/137	-	-
Kulin et al. 1998 [28]	Paroxetine Fluoxetine Sertraline Fluvoxamine	Prospective controlled cohort	31	First trimester	30/267	21/267	9/222	9/235	2/222	4/235	-	-
Nulman et al. 1997 [29]	Fluoxetine	Prospective cohort	30.5	First trimester	-	-	2/55	2/84	2/55	2/84	-	-
Chambers et al. 1996 [30]	Fluoxetine	Prospective cohort	31	First trimester	23/169	22/254	9/164	9/226	3/164	1/226	56/97	119/153
Pastuszak et al. 1993 [31]	Fluoxetine		ND	First trimester	19/128	10/128	9/28	2/110	1/98	0/110	-	-

ND: not determined, SSRI: selective serotonin reuptake inhibitor.

### Spontaneous abortion due to exposure to SSIRs in comparison to placebo

**Figure 2:**
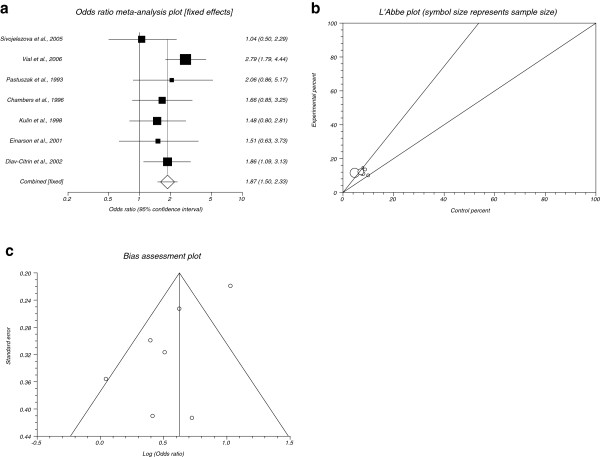
**a.Individual and summary odds ratio for the outcome of“spontaneous abortion”for studies including SSRIs exposure during pregnancy.b**. Heterogeneity indicators for the outcome of “spontaneous abortion” for studies including SSRIs exposure during pregnancy. **c**. Publication bias indicators for the outcome of “spontaneous abortion” for studies including SSRIs exposure during pregnancy.

The summary OR of spontaneous abortion for all included data for SSRIs in comparison to placebo in seven studies [20,21,25,27,28,30,31] was 1.87 with 95% CI= 1.5 to 2.33 (P< 0.0001, Figure 2a). The Cochrane Q test for heterogeneity indicated that the studies are not heterogeneous (P= 0.3113, Figure 2b) and could be combined, thus the fixed effects for individual and summary for OR was applied. Regression of normalized effect vs. precision for all included studies for spontaneous abortion among SSRIs vs. placebo therapy was 3.01 (95% CI= −6.94 to 0.924, P= 0.11) and Kendall’s test on standardized effect vs. variance indicated tau= −0.24, P= 0.38 (Figure 2c).

### Major malformations post exposure to SSIRs in comparison to placebo

**Figure 3:**
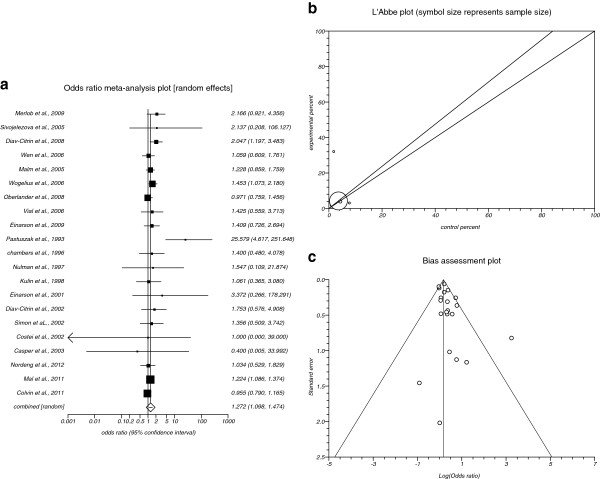
**a.Individual and summary odds ratio for the outcome of“major malformations”for studies including SSRIs exposure during pregnancy.b**. Heterogeneity indicators for the outcome of “major malformations” for studies including SSRIs exposure during pregnancy. **c**. Publication bias indicators for the outcome of “major malformations” for studies including SSRIs exposure during pregnancy

The summary OR of major malformations for all included data for SSRIs in comparison to placebo in twenty one studies [7-9,11,13-15,18-31] was 1.272 with 95% CI = 1.098 to 1.474 (P = 0.0014, Figure 3a), a significant results. The Cochrane Q test for heterogeneity indicated that the studies are heterogeneous (P = 0.0198, Figure 3b) and could not be combined, thus the random effects for individual and summary for OR was applied. Regression of normalized effect vs. precision for all included studies for major malformations among SSRIs vs. placebo therapy was 0.75 (95% CI = −0.09 to 1.52, P = 0.08) and Kendall’s test on standardized effect vs. variance indicated tau= 0.08, P = 0.66 (Figure 3c).

### Cardiac malformations post exposure to SSIRs in comparison to placebo

**Figure 4:**
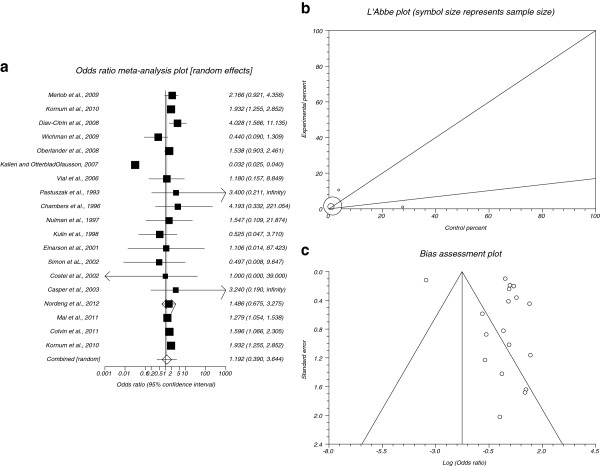
**a.Individual and summary odds ratio for the outcome of“cardiac malformations”for studies including SSRIs exposure during pregnancy.b**. Heterogeneity indicators for the outcome of “cardiac malformations” for studies including SSRIs exposure during pregnancy. **c**. Publication bias indicators for the outcome of “cardiac malformations” for studies including SSRIs exposure during pregnancy.

The summary OR of cardiac malformations for all included data for SSRIs in comparison to placebo in 19 studies [7-12,14-17,20,23,24,26-31] was 1.192 with 95% CI= 0.39 to 3.644 (Figure 4a). The Cochrane Q test for heterogeneity indicated that the studies are heterogeneous (P< 0.0001, Figure 4b) and could not be combined, thus the random effects for individual and summary for OR was applied. Regression of normalized effect vs. precision for all included studies for cardiac malformations among SSRIs vs. placebo therapy was 2.18 (95% CI= −2.71 to 7.06, P= 0.36) and Kendall’s test on standardized effect vs. variance indicated tau= −0.41, P= 0.02 (Figure 4c).

### Minor malformations post exposure to SSIRs in comparison to placebo

**Figure 5:**
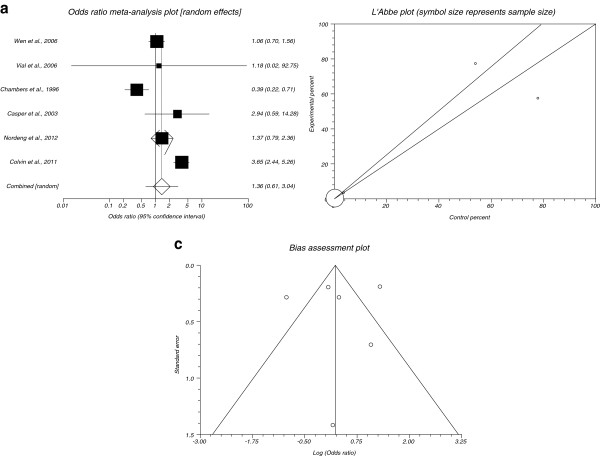
**a.Individual and summary odds ratio for the outcome of“minor malformations”for studies including SSRIs exposure during pregnancy.b**. Heterogeneity indicators for the outcome of “minor malformations” for studies including SSRIs exposure during pregnancy. **c**. Publication bias indicators for the outcome of “minor malformations” for studies including SSRIs exposure during pregnancy.

The summary OR of minor malformations for all included data for SSRIs in comparison to placebo in six studies [7,9,18,20,23,30] was 1.36 with 95% CI= 0.61 to 3.04 (Figure 5a). The Cochrane Q test for heterogeneity indicated that the studies are heterogeneous (P< 0.0001, Figure 5b) and could not be combined, thus the random effects for individual and summary for OR was applied. Regression of normalized effect vs. precision for all included studies for minor malformations among SSRIs vs. placebo therapy was −0.93 (95% CI= −9.38 to 7.52, P= 0.78) and Kendall’s test on standardized effect vs. variance indicated tau= −0.07, P= 0.72 (Figure 5c).

## Discussion

The present study was done to update our previous meta-analysis on the effects of exposure to SSRIs during pregnancy on four obstetrical outcomes including major malformations, cardiovascular malformations, minor malformations, and spontaneous abortion, to evaluate whether SSRIs increase the risk of these outcomes. The results showed that SSRIs increase the risk of spontaneous abortion and major malformations significantly but the risk of minor and cardiovascular malformations did not show significant increase. Our previous meta-analysis demonstrated only a significant increase in the risk of spontaneous abortion while in the current study the risk of major malformations was increased by SSRIs.

There are some differences between current meta-analysis and previous one. One is the number of studies included because collection of data for previous meta-analysis was up to August 2005 but for the current one was up to March 2012. In the previous meta-analysis, only 9 studies were eligible to be included while there were 25 eligible studies in the present meta-analysis. Thus the number of subjects included for each outcome is dramatically increased in the current meta-analysis. In previous meta-analysis, 2378 subjects included for spontaneous abortion whereas 4140 subjects included for this outcome in the current one; 2699 subjects for major malformations in the previous vs.1135131 in the current one; 1923 for cardiac malformation in previous vs. 1476987 in the current one; and 883 for minor malformations in the previous vs.698770 in the current one. Therefore, although the results for outcomes of spontaneous abortion, minor malformations and cardiac malformations are not apparently different from previous meta-analysis, the current results are more accurate and reliable. Interestingly, the number of major malformations in the current meta-analysis is significant while it was non-significant in the previous one. One explanation for this difference is the number of included subjects that is about 800-times higher than that of previous one. Though the risk of major malformations was statistically non-significant but it was clinically significant in previous meta-analysis. The addition of two new drugs to the list of SSRIs from the year 2008 named citalopram and escitalopram is a good reason to this new finding. Use of citalopram during pregnancy was associated with neural tube defects [8] and septal heart defects [32]. To obtain more convincing results, it is suggested to evaluate pregnancy outcomes after exposure to individual SSRIs not all SSRIs together especially for citalopram and escitalopram. However, there are two meta-analyses about the effect of paroxetine in congenital malformations [33]. The results of these studies have shown an increased prevalence of congenital defects and heart defects with first trimester paroxetine use.

Overall, this study demonstrates an increase risk of major malformation and spontaneous abortion following the use of SSRIs during pregnancy. This study does not show an increased risk for heart defects and minor malformations. Again we would like to emphasize to consider evaluation of SSRIs in pregnancy individually. This advice is because of different reports about congenital malformations for any of them. For example, increased risk of neural defects for citalopram [8] or increased prevalence of heart defects after use of paroxetine during pregnancy [34] can be emphasized. Of course it should not be forgotten that clinical study in pregnancy and pregnant mothers has its own limitations and that is why in most of meta-analysis studies, problem of data aggregation among included studies exists [6,35-38] but they are inevitable limitations of such studies and therefore conducted meta-analyses.

## Competing interests

The authors declare no competing interests. The corresponding author Mohammad Abdollahi is Editor-in-Chief of DARU and to prevent any possible influence from his side, one of Editors of the DARU was responsible to handle external peer review process, revision, and final decision for the submitted manuscript.

## Authors’ contributions

NH did literature bibliography. RR completed literature bibliography, reviewed data and drafted some parts of the paper. SN reviewed data, conducted meta-analysis, drafted and edited the paper. MA conceived, supervised, and edited the paper. All authors read and approved the final manuscript.

## Author’s information

Shekoufeh Nikfar and Mohammad Abdollahi are members of Pregnancy and Childbirth Group in The Cochrane Database of Systematic Review that is a collaboration to provide compiled scientific systematically-reviewed evidence to aid well-informed health care decisions.
